# Mechanical, Rheological and Release Behaviors of a Poloxamer 407/Poloxamer 188/Carbopol 940 Thermosensitive Composite Hydrogel

**DOI:** 10.3390/molecules181012415

**Published:** 2013-10-08

**Authors:** Jianping Chen, Rong Zhou, Lin Li, Bing Li, Xia Zhang, Jianyu Su

**Affiliations:** College of Light Industry and Food Sciences, South China University of Technology, Guangzhou 510640, Guangdong, China; E-Mails: cjp516555989@126.com (J.P.C.); zhourong0521@126.com (R.Z.); fenlinli@scut.edu.cn (L.L.); bli@scut.edu.cn (B.L.); z.xia.scut@gmail.com (X. Z.)

**Keywords:** thermosensitive composite hydrogel, phase transition temperature, mechanical properties, rheological properties, release behavior

## Abstract

The aims of this study were to prepare a thermosensitive composite hydrogel (TCH) by mixing 24% (w/v) poloxamer 407 (P407), 16% (w/v) poloxamer 188 (P188) and 0.1% (w/v) carbopol 940 (C940), and to determine the effect of natural borneol/ (2-hydroxypropyl)-*β*-cyclodextrin (NB/HP-*β*-CD) inclusion complex on the phase transition temperature, mechanical, rheological properties, and release behaviors of the TCH using the tube inversion method, a texture analyzer, a rheometer, and *in vitro* release , respectively. The results showed that as the concentration of NB/HP-β-CD increased, the phase transition temperature of the TCH was increased from 37.26 to 38.34 °C and the mechanical properties of the TCH showed that the hardness, cohesiveness, strength, and adhesiveness were increased from 0.025 to 0.064 kg, 0.022 to 0.064 kg, 0.110 to 0.307 kg and 0.036 to 0.105 kg, respectively, but the rheological properties of the TCH showed that G′, G′′ and η were decreased from 7,760 to 157.50 Pa, 1,274 to 36.28 Pa and 1,252 to 25.37 Pas, respectively. The *in vitro* release showed that an increasing NB/HP-β-CD concentration decreased the release rate of NB from the TCH, but the amount of NB released was more than 96% at 60 min, which showed the TCH had good release behavior.

## 1. Introduction

Hydrogels are polymeric materials containing a large number of hydrophilic groups capable of holding large amounts of water in their three-dimensional networks [1]. In the swollen state, hydrogels are soft and rubbery and exhibit excellent water affinity, high thermal mechanical stabilities, and well biocompatibility [2] which makes them compounds of great interest in the chemical, medicine, pharmaceuticals, food and agriculture fields [3,4,5,6].

Over the past few years, the use of thermosensitive hydrogels, which can undergo sol–gel transitions upon heating or cooling because of changes in the intermolecular interactions such as ionic, hydrogen bonding and hydrophobic forces, is of interest and has attracted the attention of many investigators due to their potential applications as delivery matrices for drugs or cells in the biomedical engineering field. Meanwhile, various temperature-responsive materials have been developed. Poloxamer 407 (P407) is a triblock copolymer with a central hydrophobic chain of polyoxypropylene (PPO) and two identical lateral hydrophilic chains of polyoxyethylene (PEO). Due to its unique thermo-reversible gelation properties, P407 has become one of the most extensively investigated temperature-responsive materials [7]. The phase transition temperature of P407 mainly depends on its concentration [8]. However, an aqueous solution of P407 at a concentration higher than 20% forms non-chemically cross-linked hydrogels upon warming to ambient temperature. P407 solution at such a concentration has a lower phase transition temperature (<25 °C), thus P407 solutions would become gels at room temperature and are thus difficult to use for drug delivery [8]. To develop a temperature-responsive gel with a suitable phase transition temperature for transdermal drug delivery system, Wei *et al*. incorporated poloxamer 188 (P188) into P407 solutions to modulate the phase transition temperature [9]. The phase transition temperatures of the poloxamer mixture solutions were higher than those of the individual P407 solutions. In addition, P407 and P188 are known to have low toxicity, excellent water-solubility, high solubilizing capacity for acetaminophen, good drug release characteristics, compatibility with other chemicals and to cause less skin irritation, but the P407/P188 thermosentive hydrogel cannot meet the needs for clinical application because of its low gel strength. Moreover, studies on improving the gel strength of P188/P407 thermosentive hydrogel are scarse.

As a synthetic polymer, carbopol has been often used recently as a component of drug delivery systems [10]. Owing to its high viscosity, therefore, in this work, carbopol 940 (C940) was selected as the bioadhesive polymer to reinforce the gel strength of the P407/P188 thermosentive hydrogel. P407, P188 and C940 were used to prepare a thermosensitive composite hydrogel (TCH). Due to its good stability and solubility, natural borneol/(2-hydroxypropyl)-*β*-cyclodextrin (NB/HP-*β*-CD) prepared in our previous study [11] was chosen as a model drug. In order to determine the effect of NB/HP-*β*-CD concentration on TCH, the phase transition temperature, mechanical properties and rheological properties of the TCH were investigated using the tube inversion method, a texture analyzer and a rheometer, respectively. Furthermore, the release behaviors of the TCH were also evaluated *in vitro*.

## 2. Results and Discussion

### 2.1. Characterization of TCH

#### 2.1.1. Phase Transition Temperature

Phase transition temperature is the temperature at which the liquid phase undergoes a transition into a hydrogel. An ideal hydrogel should be a free flowing liquid at room temperature, transform into a hydrogel at phase transition temperature, and sustain drug release under physiological conditions. Poloxamers are commonly used materials for *in situ* hydrogel formation. They are ABA-type triblock copolymers composed of polyethylene oxide (PEO) and polypropylene oxide (PPO) units. As the temperature increases, dehydration of the PPO leads to formation of a micelle core while hydration of the PEO causes it to expand and form an outer skin. As the temperature continues to rise, the micelles arrange themselves in sequence to form a hydrogel. Typically, the phase transition temperature depends on the PEO:PPO ratio in the polymer solution [12]. The phase transition temperatures of F_0_, F_1_, F_2_ and F_3_ determined by the tube inversion method are shown in Table 1. Typically, the phase transition temperature is between 30 and 40 °C, which is suitable for a transdermal drug delivery system. If the phase transition temperature is lower than 30 °C, it can easily form gels at the room temperature and it will be difficult to use for drug delivery [8], however, if the phase transition temperature is higher than 40 °C, it is difficult to form gels. According to the results, with the concentrations of NB/HP-*β*-CD inclusion complex increasing from 0.1 to 0.3%, the phase transition temperature of TCH slightly increased from 37.26 to 38.34 °C, indicating it has a suitable transition temperature for transdermal drug delivery system. It was speculated that NB/HP-*β*-CD inserted into the poloxamer chains might disturb the micelles packing and entanglements of P407 and P188 [13]. In addition, the different of concentrations of NB/HP-*β*-CD resulted in the difference in PEO and PPO ratio, which depended on the phase transition temperature, and the increase of the NB/HP-*β*-CD concentration did not affect the gelling process of TCH.

**Table 1:** The effect of NB/HP-β-CD concentration of inclusion complex on thermosensitive composite hydrogel.

Formulation	hydrogel temperature (°C)
F_0_	37.26 ± 0.26
F_1_	37.52 ± 0.35
F_2_	37.70 ± 0.03
F_3_	38.34 ± 0.15

F_0_: Thermosensitive composite hydrogel. F_1_: Thermosensitive composite hydrogel adding 1% NB/HP-β-CD. F_2_: Thermosensitive composite hydrogel adding 2% NB/HP-β-CD. F_3_: Thermosensitive composite hydrogel adding 3% NB/HP-β-CD.

#### 2.1.2. Hydrogel Texture

**Figure 1:**
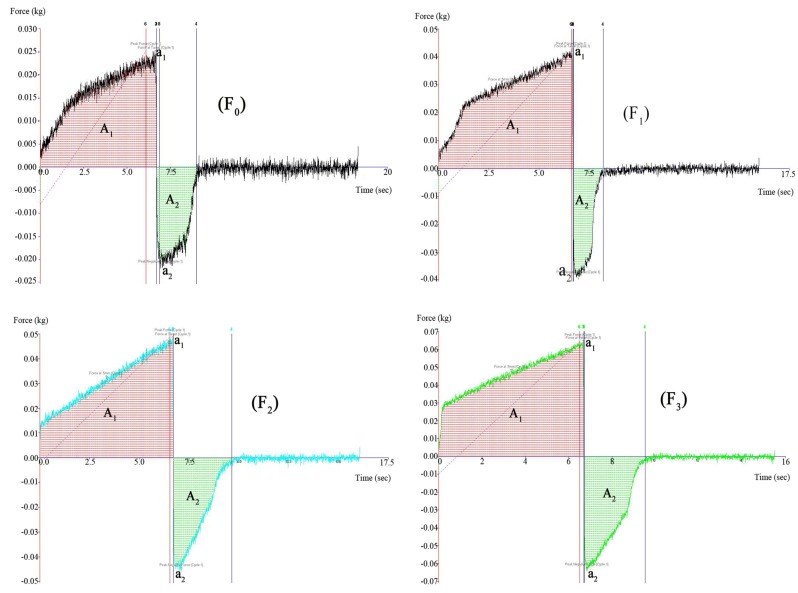
The effect of NB/HP-β-CD concentration on hardness, cohesiveness, strength and adhesiveness of thermosensitive composite hydrogel (F_0_: Thermosensitive composite hydrogel; F_1_: Thermosensitive composite hydrogel adding 1% NB/HP-β-CD; F_2_: Thermosensitive composite hydrogel adding 2% NB/HP-β-CD; F_3_: Thermosensitive composite hydrogel adding 3% NB/HP-β-CD).

Texture profile analysis (TPA) was originally proposed as a suitable method to characterize semisolid drug dosage forms by Jones and his group [14]. TPA could provide a reliable overview of those mechanical properties of hydrogel. Hydrogel texture properties are known to depend on the composition and concentration of hydrogels. The mechanical properties of the TCH in the presence of various concentrations of NB/HP-*β*-CD were shown below (Table 2 and Figure 1). Table 2 and Figure 1 reveal that as the concentration of NB/HP-*β*-CD increased, the hardness, cohesiveness, strength, and adhesiveness of the TCH were increased from 0.025 to 0.064 kg, 0.022 to 0.064 kg, 0.110 to 0.307 kg and 0.036 to 0.105 kg, respectively.

**Table 2:** The effect of NB/HP-β-CD concentration on mechanical properties of thermosensitive composite hydrogel.

Formulation	Hardness (a_1_)/(Kg)	Cohesiveness (a_2_)/(Kg)	Strength (A_1_)/(Kg)	Adhesiveness (A_2_)/(Kg)
F_0_	0.025	0.022	0.110	0.036
F_1_	0.042	0.040	0.189	0.037
F_2_	0.049	0.046	0.207	0.070
F_3_	0.064	0.064	0.307	0.105

F_0_: Thermosensitive composite hydrogel. F_1_: Thermosensitive composite hydrogel adding 1% NB/HP-β-CD. F_2_: Thermosensitive composite hydrogel adding 2% NB/HP-β-CD. F_3_: Thermosensitive composite hydrogel adding 3% NB/HP-β-CD.

The mechanical property-enhancing effects of NB/HP-*β*-CD assume that inserted into the poloxamer chains it might disturb the micelle packing and poloxamer entanglements. For all the formulations, the hardness was lower than 0.1 kg and the strength was higher than 0.1 kg, which indicated that the TCH had low hardness and high strength. The low hardness and high strength reflects the applicability of the TCH in the further clinical application.

#### 2.1.3. Rheological Properties

**Figure 2:**
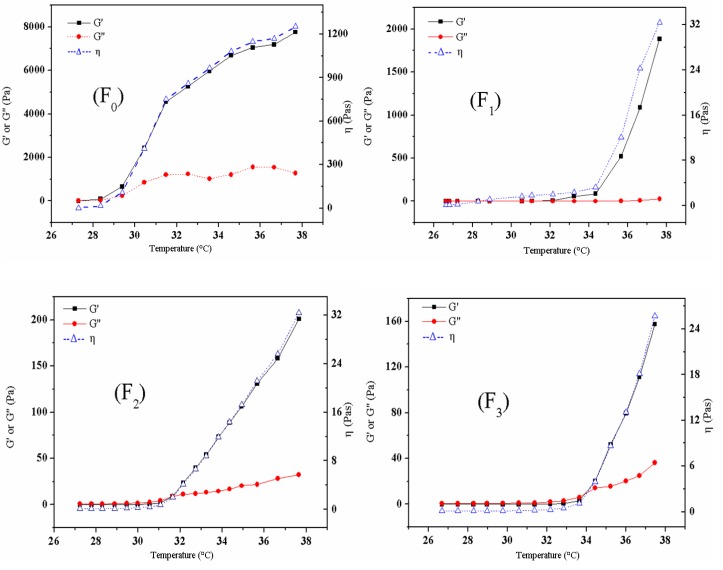
The effect of NB/HP-β-CD concentration on rheological properties of thermosensitive composite hydrogel (F_0_: Thermosensitive composite hydrogel; F_1_: Thermosensitive composite hydrogel adding 1% NB/HP-β-CD; F_2_: Thermosensitive composite hydrogel adding 2% NB/HP-β-CD; F_3_: Thermosensitive composite hydrogel adding 3% NB/HP-β-CD).

The relationship between elastic modulus (G′) and loss modulus (G′′) can reflect the change of viscosity and elasticity between a weak hydrogel and strong hydrogel [15]. When the G′ is lower than the G′′, the material behaves like a viscous liquid, otherwise, it like a hydrogel with elastic behavior. From the viewpoint of rheology, a high G′ is one of the inherent characteristics of solid materials, and the process of a phase change from liquid to semisolid can be described via the increment in G′ [9]. Figure 2 illustrates the rheological properties of aqueous hydrogels composed of a range of concentrations of NB/HP-*β*-CD (1%, 2%, 3% w/v).

It was clear from Figure 2 that for all the formulations, G′, G′′ and η of the TCH were very low at the initial temperature, which indicated it is liquid and with the temperature rose, the G′, G′′ and η were gradually increased as a result of gel formation. Moreover, in the whole range of temperatures examined, the increasing NB/HP-*β*-CD concentration decreased the G′, G′′ and η of the TCH. Specifically, for all the formulations, with the enhancement of NB/HP-β-CD concentration, the maximum value of G′, the maximum value of G′′ and the maximum value of η were gradually decreased from 7,760 (F_0_) to 1,883.39 (F_1_), 201.00 (F_2_), 157.50 ( F_3_), 1,274 (F_0_) to 23.85 (F_1_), 32.30 (F_2_), 36.28 (F_3_) and 1252 (F_0_) to 32.53 (F_1_), 32.39 (F_2_), 25.37 (F_3_), respectively (see Table 3). Interestingly, as Figure 2 showed, G′ and η value of F_1_ was higher than those of F_2_ and F_3_, while G′′ value of F_1_ was slightly lower than those of F_2_ and F_3_ at the same temperature. It was speculated that the elastic modulus decrease might be related with the changes of the PEO and PPO ratio because the increasing NB/ HP-*β*-CD concentration resulted in a difference in PEO and PPO ratio. Meanwhile, for each formulation, G′ was lower than G′′ before the crossover and then higher than G′′ after the crossover. It was likely that at lower temperature, the formation of macromolecule chain entanglements is difficult, so the loss modulus increases more quickly than the elastic modulus does until the two curves cross over. After crossover, the elastic modulus dominates the loss modulus. Moreover, for each formulation, G′ was increased drastically with increasing temperature. It was likely that as temperature increases, poloxamer copolymer molecules aggregate into micelles due to the dehydration of hydrophobic PO blocks of the poloxamer [16]. The ordered packing of micelles formed the hydrogel. In addition, there are more entanglements and aggregates among macromolecular chains in the solution at high temperature.

**Table 3:** The effect of NB/HP-β-CD concentration on rheological properties of thermosensitive composite hydrogel.

Formulation	G′ max/(Pa)	G′′max/(Pa)	ηmax/(Pas)
F_0_	7,760.00	1,274.00	1,252.00
F_1_	1,883.89	23.85	32.53
F_2_	201.00	32.30	32.39
F_3_	157.50	36.28	25.73

F_0_: Thermosensitive composite hydrogel. F_1_: Thermosensitive composite hydrogel adding 1% NB/HP-β-CD. F_2_: Thermosensitive composite hydrogel adding 2% NB/HP-β-CD. F_3_: Thermosensitive composite hydrogel adding 3% NB/HP-β-CD.

### 2.2. *In-vitro* Release

**Figure 3:**
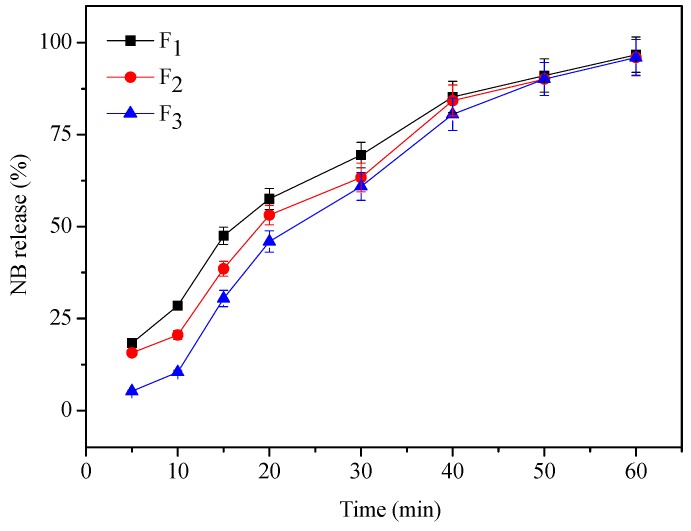
The effect of NB/HP-β-CD on the release profileof NB from thermosensitive composite hydrogel (*n* = 3) (F_1_: Thermosensitive composite hydrogel adding 1% NB/HP-β-CD; F_2_: Thermosensitive composite hydrogel adding 2% NB/HP-β-CD; F_3_: Thermosensitive composite hydrogel adding 3% NB/HP-β-CD).

To examine whether NB/HP-*β*-CD affects the release rate of NB from TCH, we performed the release test with F_1_, F_2_ and F_3_. The release amount of NB from F_1_, F_2_ and F_3_ was measured by gas chromatography (Agilent N9890, Agilent Technologies, Palo Alto, CA, USA). The temperature 37 ± 0.5 °C was kept constant. Figure 3 showed the cumulative amount of NB released *vs.* time profiles for F_1_, F_2_ and F_3_. The results showed that at 5 min, significant differences were observed among F_1_, F_2_ and F_3_. Moreover, F_1_ showed the fastest release of NB. F_2_ and F_3_ showed similar release patterns whereas F_3_ revealed the slowest release. All the formulations (F_1_, F_2_ and F_3_) released more than 40% NB at 20 min and 60% at 30 min. From 60 min onwards, there was no significant difference among the formulations and the cumulative amount of NB release from TCH was more than 96%, which demonstrated that TCH displayed good release behaviors. Increasing the NB/HP-*β*-CD concentration significantly lowered the release rate of NB and produced sustained release of NB by a mechanism involving thermosensitive gel corrosion and prolonged the release time of NB, which met the needs for transdermal drug delivery system. These results revealed that this TCH system could be promising in prolonged topical drug dosage administration.

## 3. Experimental

### 3.1. Materials

NB/HP-*β*-CD inclusion complex was prepared according to our previous study [11]. P407, P188 and C940 were purchased from Yuanpeng Biological Technology Co., Ltd., Jinan, China. All other chemicals and solvents were of analytical grade and purchased from China National Medicine Co., Ltd., Beijing, China

### 3.2. Preparation of TCH

Hydrogels were prepared according to Park *et al*. [17]. In brief, P407, P188, C940, NB/HP-*β*-CD were solubilized in distilled water and left at 4 °C for 24 h until a clear solution was obtained. The compositions of the various formulations are listed in Table 4. As listed in Table 4, a certain amount of P407, P188, C940, NB/HP-*β*-CD solutions were mixed in five 20 mL transparent beakers to prepare four samples which were denoted as solutions F_0_, F_1_, F_2_, F_3_, respectively. Then, each 20 mL transparent beaker was kept in a 40 °C DZKW-S-4 constant-temperature thermostat water bath (Yongguangming Medical Instrument Company, Beijing, China) for 10 min until the gelling process was completed.

**Table 4:** Composition of hydrogels.

Ingredients (w/v)	F_0_ (%)	F_1_ (%)	F_2_ (%)	F_3_ (%)
P407	24	24	24	24
P188	16	16	16	16
C940	0.1	0.1	0.1	0.1
NB/HP-β-CD	0	1	2	3

### 3.3. Characterization of TCH

#### 3.3.1. Measurement of Phase Transition Temperature

The phase transition temperature of the TCH was measured by the tube inversion method [18,19], *i.e.*, F_0_, F_1_, F_2_ and F_3_ solutions were placed in four DZKW-S-4 constant-temperature thermostat water baths (Yongguangming Medical Instrument Company), respectively. Four digital thermosensors connected to thermistor were immersed in the four mixed solutions, respectively. Each solution was heated at a rate of 2 °C every 5 min. When the polymer solution stopped flowing after a beaker inversion due to gelation, the temperature displayed on the thermistor was determined as a phase transition temperature [20]. The following tests were performed in triplicate.

#### 3.3.2. Hydrogel Texture

The texture properties of the TCH were performed using a Texture Analyser TA.XT Plus (SMS Ltd., Godalming, UK) as previously described [21,22]. In order to avoid air into the samples and assure formation of a smooth upper surface, 15 mL of F_0_, F_1_, F_2_, and F_3_ solutions were filled in four standard penicillin bottles (20 mL), respectively. When each solution transformed to hydrogel, a 10-mm (diameter) disk was compressed into the hydrogel and redrawn. The speed rate and distance (depth of the insertion) were 1.5 mm/s and 10 mm, respectively. Hydrogel parameters such as hardness, cohesiveness, strength and adhesiveness were determined from the resultant force-time plot (Figure 1). The maximum force (a_1_) presents the hardness of the TCH, which is defined as the force required to attain a given deformation, and cohesiveness (a_2_) is defined as the work required to deform the hydrogel in the downwards movement of the probe. The first area (A_1_) shows the strength of the hydrogel to the probe, while the second area (A_2_) shows the adhesiveness of the hydrogel to the probe [21,22].

#### 3.3.3. Rheological Properties of the Hydrogels

The rheological tests were carried out with a Haake MARS III rotational rheometer (Thermo Fisher Scientific, Schwerte, Germany) using a Peltier unit to control the temperature. In the oscillatory mode, a parallel plate (40 mm diameter, PP35Ti) geometry measuring system was employed, and the gap was set to 1 mm. After the sample was correctly placed in the Peltier plate, all the tests were started at 26 ± 0.1 °C. The test was performed to outline sample behaviour at constant frequency (1 Hz). The temperature was increased from 26 to 38 °C at a rate of 2 °C/min. The storage modulus (G′), the loss modulus (G′′) and the dynamic viscosity (η) were recorded.

### 3.4. *In-Vitro* Release

The *in vitro* release of NB was determined from different hydrogel formulations (F_1_, F_2_, F_3_ solutions) placed in penicillin bottles under constant shaking. Two mL of each hydrogel formulation was packed into the 20-mL penicillin bottle. These penicillin bottles were placed in a water bath (37.5 °C) until hydrogel formed. The release medium was 2 mL of normal saline (0.9% NaCl) providing sink conditions for NB. The medium was maintained at 37.5 °C and shaken at 80 rpm. At various time intervals, 2 mL of dissolution fluid was collected. Content of NB in the samples was analyzed by gas chromatography (Agilent N9890). The exact amount of NB released from the formulation was calculated with a calibration curve of NB according to the equation: y = 1307x + 10.14, R = 0.9994. Each experiment was performed in triplicate.

### 3.5. Determination of the Calibration Curve of NB

#### 3.5.1. Gas Chromatography (GC)

Quantitative analysis of NB was performed using a gas chromatography (Agilent N9890) with a split/splitless injection port and a flame ionization detection system (FID), under the following operational conditions: column HP-5MS fused silica capillary column (30 m × 0.25 mm i.d.; 0.25 μm). Pure nitrogen (99.999%) was used as the carrier gas at a constant flow of 7.8 mL/min, and an injection volume of 1 μL was employed with an injector temperature of 250 °C. The oven temperature was initiated at 100 °C (held for 1 min), then raised at the rate of 10 °C/min to 170 °C, and held for 5 min at this temperature. The detector and column temperatures were 270 °C and 100 °C, respectively.

#### 3.5.2. Measurement of the Calibration Curve of NB

NB (250 mg) was dissolved in anhydrous ethanol (50 mL). Then, 0.5, 1.0, 1.5, 2.0, 2.5, 3.0 and 3.5 mL solutions were taken out and diluted to 10 mL with anhydrous ethanol, respectively. These samples were analyzed by GC. The concentration (x) and the peak area (y) of NB had a good relationship as follows:

y = 1307x + 10.14, R = 0.9994
(1)

### 3.6. Statistical Analysis

The obtained data were expressed as the mean ± standard deviation of triplicate determinations. Statistical analysis was performed using the software SPSS 15.0 (SPSS, Chicago, IL, USA).

## 4. Conclusions

A TCH based on poloxamer 407 (P407), poloxamer 188 (P188) and carbopol 940 (C940) was prepared, and the effects of NB/HP-*β*-CD complex concentration on TCH was also investigated. The results showed that in the whole range of the concentration of NB/HP-*β*-CD examined, TCH still exhibited a suitable phase temperature with 37.26–38.34 °C, and suitable TCH mechanical behaviors with low hardness (0.025–0.064 kg) and high strength (0.110–0.307 kg), and desired release behaviors with more than 96% NB released from TCH at 60 min. Moreover, the increasing of the concentration of NB/HP-*β*-CD decreased its rheological properties. Thus, the TCH should be applied as a transdermal delivery system for NB. Although TCH appears to be a promising new vehicle for transdermal drug delivery, additional studies will be required before gel formulations can be developed for specific therapeutic agents. This work indicated the TCH had application potential for other drugs and provided valuable information for studying the dosage of NB/HP-*β*-CD.
